# Supplementary material for: Fludarabine, High-Dose Cytarabine and Idarubicin-Based Induction May Overcome the Negative Prognostic Impact of FLT3-ITD in NPM1 Mutated AML, Irrespectively of FLT3-ITD Allelic Burden
Source: Cancers (Basel). 2020 Dec 24;13(1):34. doi: 10.3390/cancers13010034 (PMC7796342; doi:10.3390/cancers13010034)
Supplement: Supplementary file 1 [file cancers-13-00034-s001.pdf]

Fludarabine, High-Dose Cytarabine and Idarubicin-Based In-duction May Overcome the Negative Prognostic Impact of Flt3-Itd Innpm1 Mutated AML, Irrespectively of Flt3-Itd Allelic Burden

Paola Minetto, Anna Candoni, Fabio Guolo, Marino Clavio, Maria Elena Zannier, Maurizio Miglino, Maria Vittoria Dubbini, Enrico Carminati, Anna Sicuranza, Sara Ciofini, Nicoletta Colombo, Girolamo Pugliese, Riccardo Marcolin, Adele Santoni, Filippo Ballerini, Luca Lanino, Michele Cea, Marco Gobbi, Monica Bocchia, Renato Fanin and Roberto Massimo Lemoli

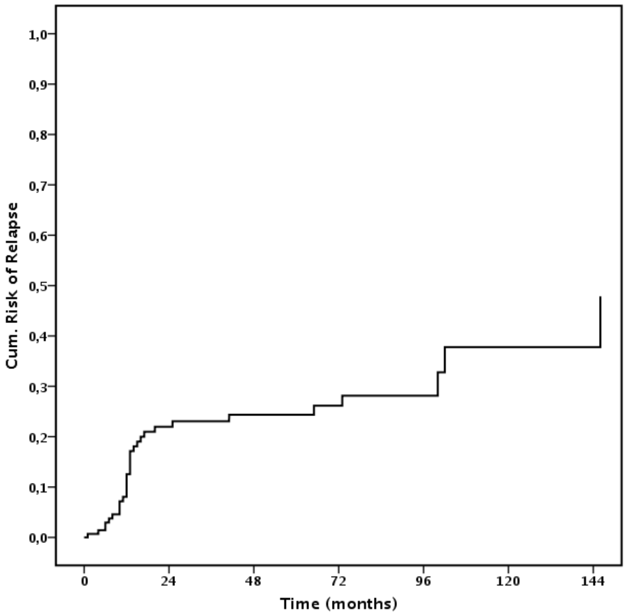

Figure S1. Cumulative Incidence of Relapse in all patients.

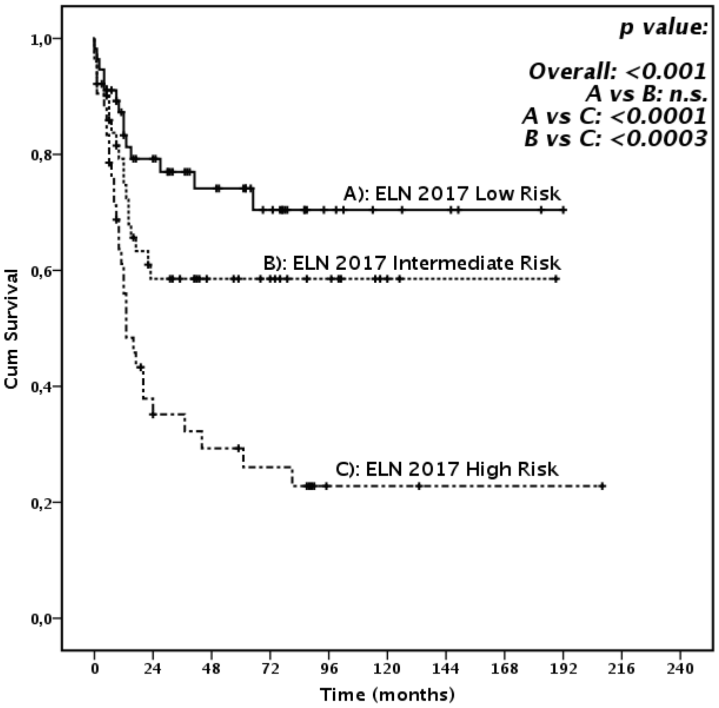

Figure S2. Overall Survival in all patients according to ELN 2017.

© 2020 by the authors. Licensee MDPI, Basel, Switzerland. This article is an open access article distributed under the terms and conditions of the Creative Commons Attribution (CC BY) license (http://creativecommons.org/licenses/by/4.0/).
